# Supplementary material for: Studies on Anion Exchange Membrane and Interface Properties by Electrochemical Impedance Spectroscopy: The Role of pH
Source: Membranes (Basel). 2021 Oct 10;11(10):771. doi: 10.3390/membranes11100771 (PMC8540937; doi:10.3390/membranes11100771)
Supplement: Supplementary file 1 [file membranes-11-00771-s001.zip › membranes-1370891-supplementary.pdf]

membranes

Supporting information

Studies on Anion Exchange Membrane and Interface Properties by Electrochemical Impedance Spectroscopy: The Role of pH

Wenjuan Zhang [1,*], Wei Cheng [2], Ramato Ashu Tufa [3], Caihong Liu [4,*], David Aili [3],Debabrata Chanda [5], Jing Chang [1],Shaopo Wang [1], Yufeng Zhang [1] and Jun Ma [6]

1 Tianjin Key Laboratory of Aquatic Science and Technology, School of Environmental and Municipal Engineering, Tianjin Chengjian University, Tianjin 300384, China; changjinghit@163.com (J.C.); wspfr@sina.com (S.W.); zyf9182@163.com (Y.Z.)
2 Research Center for Eco-Environmental Sciences, Chinese Academy of Sciences, Beijing 100085, China; weicheng@rcees.ac.cn
3 Department of Energy Conversion and Storage, Technical University of Denmark, Building 310, 2800 Kgs. Lyngby, Denmark; rastu@dtu.dk (R.A.T.); larda@dtu.dk (D.A.)
4 Key Laboratory of Eco-Environments in Three Gorges Reservoir Region, College of Environment and Ecology, Chongqing University, Ministry of Education, Chongqing 400044, China
5 College of Chemistry and Chemical Engineering, Henan University, Kaifeng 475004, China; dchanda32@gmail.com
6 State Key Laboratory of Urban Water Resource and Environment, Harbin Institute of Technology, Harbin 150090, China; majunhit@163.com
* Correspondence: wenjuanvivian@126.com (W.Z.); lchhit@126.com (C.L.); Tel./Fax: +86-22-23085117 (W.Z.)

1. Solution compositions and pH

The compositions and pH for the test solutions are shown in Table S1. The pH of the solution was adjusted by adding HCl or NaOH.

Table S1. The compositions and pH for the test solutions.

Sample	pH	NaCl (M)	HCl (M)	NaOH (M)
1	2.11		0.01	—
2	3.05		0.001	—
3	6.26	0.5	—	—
4	10.94		—	0.001
5	12.15		—	0.01

2. Ion exchange capacity (IEC) and membrane swelling

Following the argentometric titration, the IEC was calculated as:

$$IEC_A = \frac{(V_b - V_{p1})C_{AgNO_3}}{m_{dry}} \quad \text{(S1)}$$

where IEC_A (meq/g) is the IEC of anion exchange membrane, V_b (mL) is the consumped volume of $AgNO_3$ for blank titration, V_{p1} (mL) is the consumed volume of $AgNO_3$ at the first equivalent point, C_{AgNO_3} is the concentration of $AgNO_3$ (0.1mol/L), and m_{dry} is the dry weight of membrane sample;

For the method based-on acid-base titrations, the IEC was calculated as:

$$IEC_C = \frac{(V_b - V_p)C_{NaOH}}{m_{dry}} \quad \text{(S2)}$$

where IEC_C (meq/g) is the IEC of cation exchange membrane, V_b (mL) is the consumped volume of NaOH for blank titration, V_p (mL) is the consumped volume of NaOH at the equivalent point, C_{NaOH} is the concentration of NaOH (0.1mol/L).

The swelling degree was calculated by the following formula:

$$sw\ (\%) = \frac{m_{wet} - m_{dry}}{m_{dry}} \times 100 \quad (S3)$$

where m_{wet} and m_{dry} are the weight of membranes in wet and dry conditions respectively.

The density of fixed charge groups (C_{fix}, mol/L), is defined as moles of fixed charge groups per volume of water in membrane, can be determined from the swelling degree and the IEC as follows:

$$C_{fix} = \frac{IEC}{sw\%} \times 100 \quad (S4)$$

3. FT-IR analysis for two cation exchange membranes

The transmittance FT-IR spectra of the AEMs are presented in Fig. S1. The band ~3312 cm^{-1} is due to the interchain hydrogen-bonded N-H band between imine and amine groups. The absorption peaks at 2917 cm^{-1} and 2849 cm^{-1} are attributed to C-H stretching vibration peaks on $-CH_2-$ and $-CH_3$ groups, respectively. The peaks at ~1656 cm^{-1} and 1536 cm^{-1} are ascribed to C=O and C=N groups, respectively [1]. The peak observed around 1040 cm^{-1} can be assigned to the SO_3^-, which proved that sulfonic acid groups were contained in the two cation exchange membranes (CEM-Type I and II) as fixed charges.

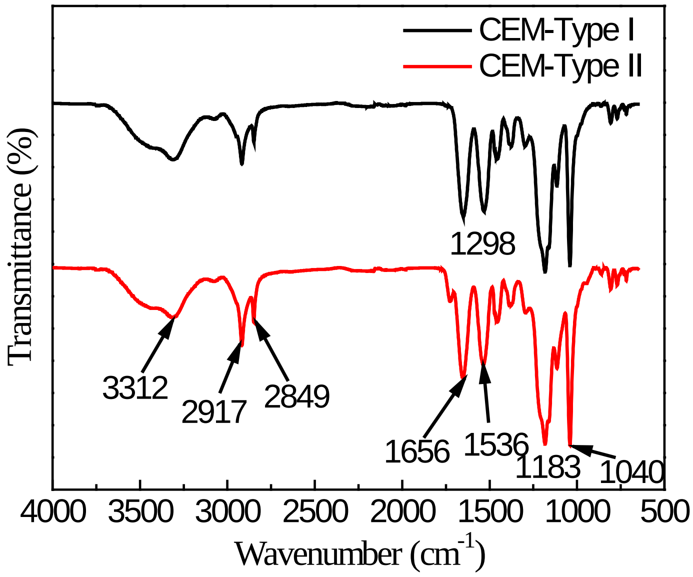

Figure S1. FT-IR spectra of two cation exchange membrane: CEM-Type I and CEM-Type II.

4. XPS analysis for two cation exchange membranes

Examination of the survey XPS spectra of four IEMs (shown in Fig. S2a) indicated the presence of carbon, oxygen, nitrogen and chlorine in two AEMs, as well as the presence of carbon, oxygen, nitrogen and sulfur in two CEMs. The fitted high resolution spectra for sulfur S2p related to the chemical bonding of CEM-Type I is displayed as an example in Fig. S2b. The atomic concentration percentages of two CEMs are listed in Table S2. It was found that the sulfur percentage on the surface of two cation exchange membranes was similar [2].

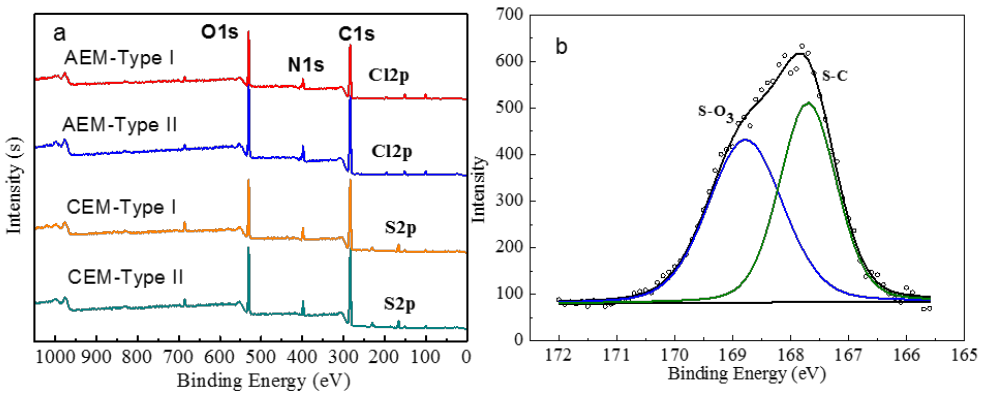

Figure S2. Survey XPS spectra from the top 1–5 nm depth of the surface region of four cation exchange membranes and the high resolution spectra and curve fitting of S2p of CEM-Type I.

Table S2. Surface atomic composition percentages of two cation exchange membranes from XPS analysis.

Membrane	Atomic composition percentage, (A.C.%)			
	Carbon (C1s)	Oxygen (O1s)	Nitrogen (N1s)	Sulfur (S2p)
CEM-Type I	69.6	21.7	6.0	2.7
CEM-Type II	68.1	22.7	6.4	2.7

5. Variations of atomatic compositions of N and S element in fixed charge groups with pH

As mentioned above, the fixed charge groups in AEMs are quaternary ammonium groups and tertiary amino groups, while in CEMs are sulfonic acid groups. The automatic compositions (A.C.%) of N and S elements in fixed charge groups of AEM and CEM, respectively can be calculated from IEC and are shown in Fig. S5. The calculated data are lower than their respective values from XPS (7.7 and 8.3 for N element in fixed charge groups of AEMs, 2.7 for S element in fixed charge groups of CEMs). It was due to the different measurement methods that the XPS spectra was obtained from the surface of membranes within the thickness of 1-10 nm, while the IEC was calculated by dividing the dry weight of the whole membrane (Eq. S1 and S2). When the fixed charge groups are in ionic form, then they own the transfer ability for counter-ions. However, the tertiary amino groups (pKa of 7~10) in AEMs can not be in charged form during the pH of 2~12 as quaternary ammonium groups (pKa > 15) and sulfonic acid groups (pKa of 0~1), that's why the A.C.% of N element in AEMs decreased with the pH increase from 2 to 12. This decrease phenomenon was more significant in AEM-Type II than AEM-Type I, which was attributed to the amount of tertiary amino groups (5.4% for AEM-Type I, 6.2% for AEM-Type II).

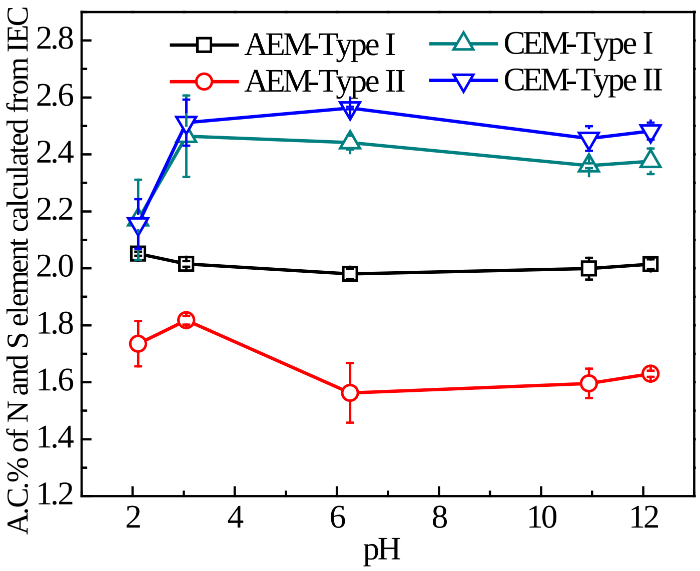

Figure S3. A.C.% of N and S element in fixed charge groups calculated from IEC.

6. Electrochemical impedance spectroscopy (EIS)

The impedance spectra of AEM-Type II with different pH are shown in Figure S4.

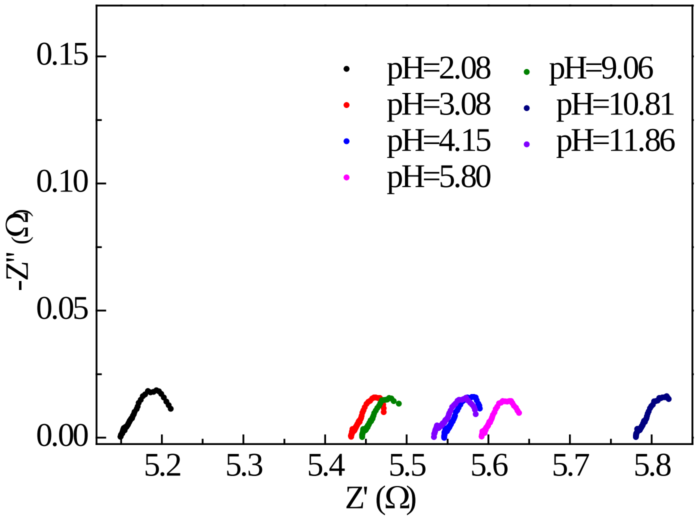

Figure S4. Impedance spectra of AEM-Type II at different pH.

Resistance of two ion-exchange membranes in 0.5 M NaCl solution at pH of 5.8 was determined from EIS measurements. Fig. S5 showed the fittings of AEM-Type I and CEM-Type I with Nyquist plots (Fig. S5a, c) and Bode plots (Fig. S5b, d) obtained from EIS measurements in 0.5 M NaCl solution at 20 ± 2 ℃ and 1 cm/s with pH of 5.8. The equivalent circuit used for fitting was obtained from the literature [2], in which the resistance of the electrical double layer and diffusion boundary layer was represented by a resistor and its capacitance was represented by a capacitor. As shown in Fig. S5, the fitting results are in good agreement with the experimental data.

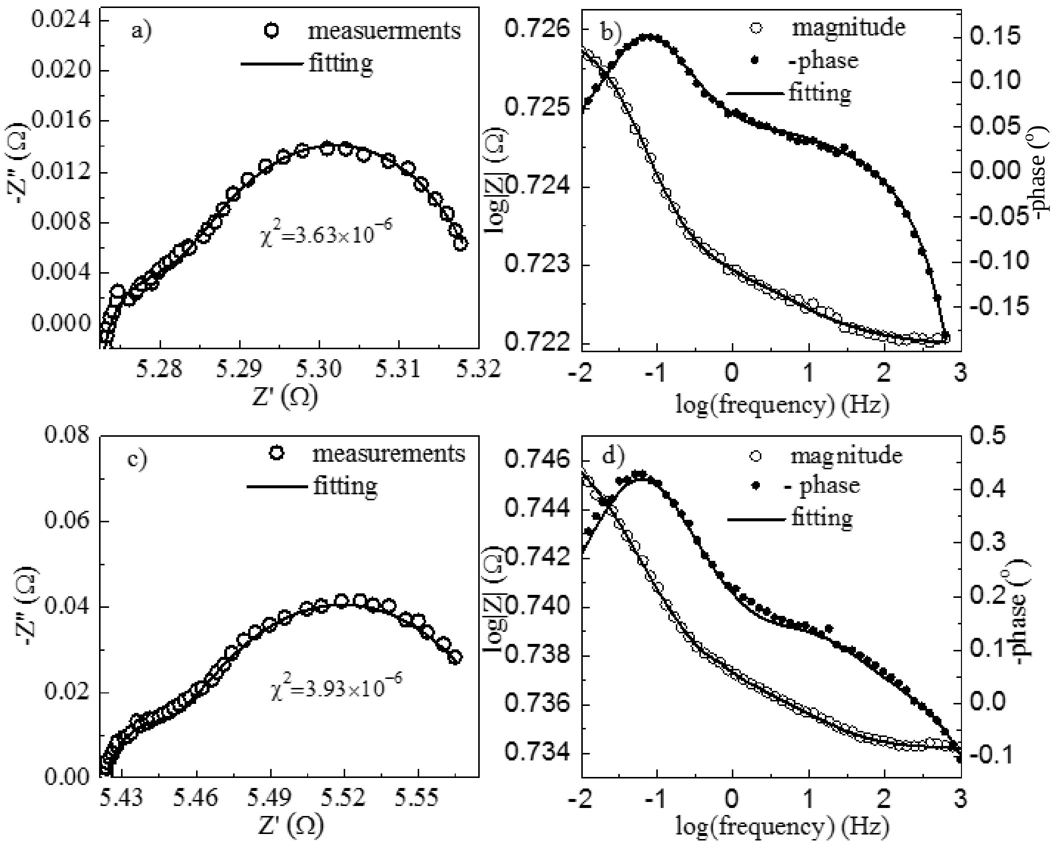

Figure S5. EIS data and fitting curves of the ion exchange membranes AEM-Type I and CEM-Type I: (a) Nyquist plot of AEM-Type I, (b) Bode plot of AEM-Type I, (c) Nyquist plot of CEM-Type I and (d) Bode plot of CEM-Type I. Conditons: flow rate 1cm/s, temperature 20±2 °C, and 0.5 mol/L NaCl at pH=5.8.

7. Reaction of fixed charge groups of anion exchange membrane in alkali media

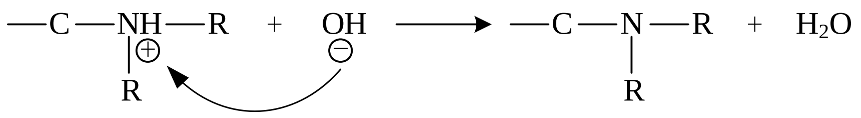

Figure S6. Reaction of a protonated tertiary amine of anion exchange membrane in alkali media[3].

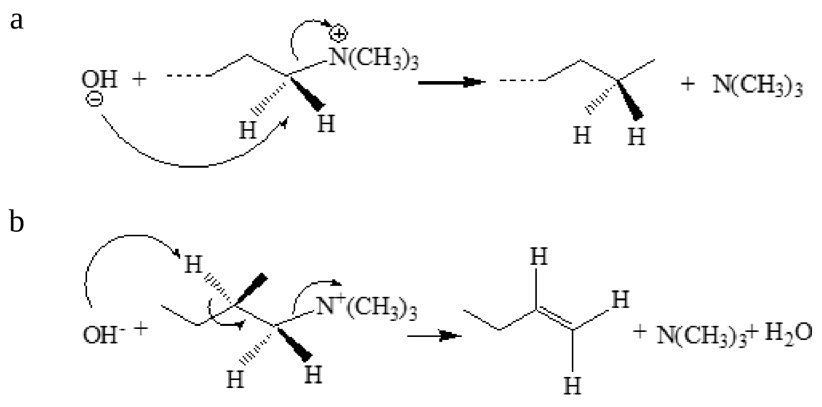

Figure S7. Degradation of the amminium groups in alkali media: a) S_N2 substitution reaction [4], b) E2 elimination reaction (Hoffman elimination reaction) [5].

8. Chemical structures of four ion exchange membranes speculated in this study

From the instrumental characterizations for identifying the functionalities for membranes investigated in this study, it can be obtained that the quaternary ammonium groups and tertiary amino groups were involved in the AEMs as fixed charge groups while the sulfonic acid groups were in the CEMs. However, these membranes are commercial membranes with no disclosed structure. Therefore, we presented the structure of the AEMs and CEMs generically as shown in Fig. S8.

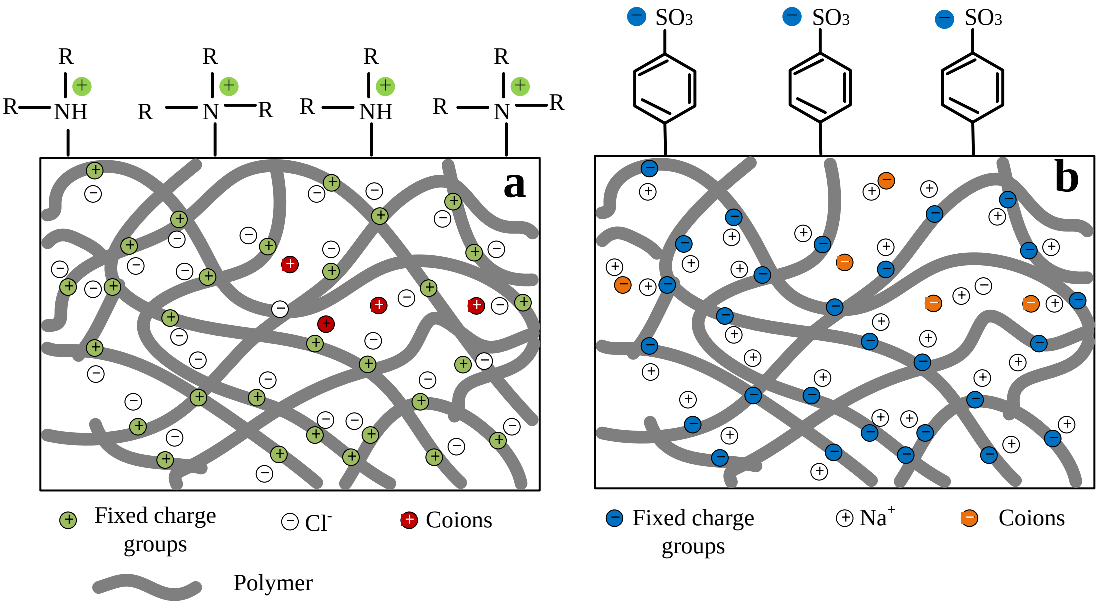

Figure S8. Generic structural presentation of the membranes with the identified functionalities: (a) anoin exchange membranes and (b) cation exchange membranes in this study. R in fixed charge groups of AEM represents alkyl, benzyl, imidazolium or benzimidazolium [6].

9. Membrane resistance and interfacial properties of cation exchange membranes before and after washing

The membrane resistance as well as resistance and capacitance of interfacial layers were shown in Fig. S9 and S10. It was observed that there was almost no variation in membrane resistance, resistance and capacitance of EDL and DBL after immersed in acid and base for three days and washed with NaCl, which indicated that there was no change in fixed charge groups and membrane structures for cation exchange membranes with sulfonic acid groups.

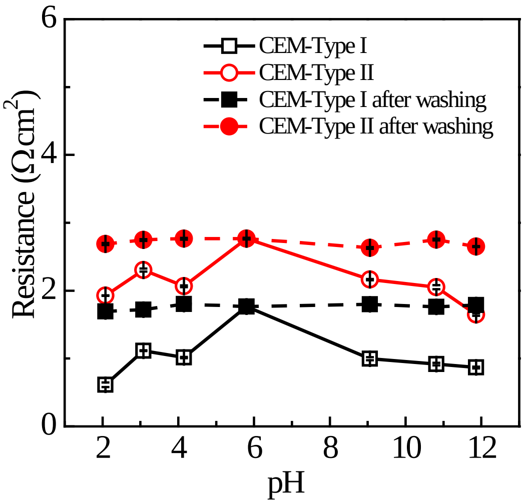

Figure S9. Membrane resistance of cation exchange membranes before and after washing with 0.5 mol/L NaCl solution.

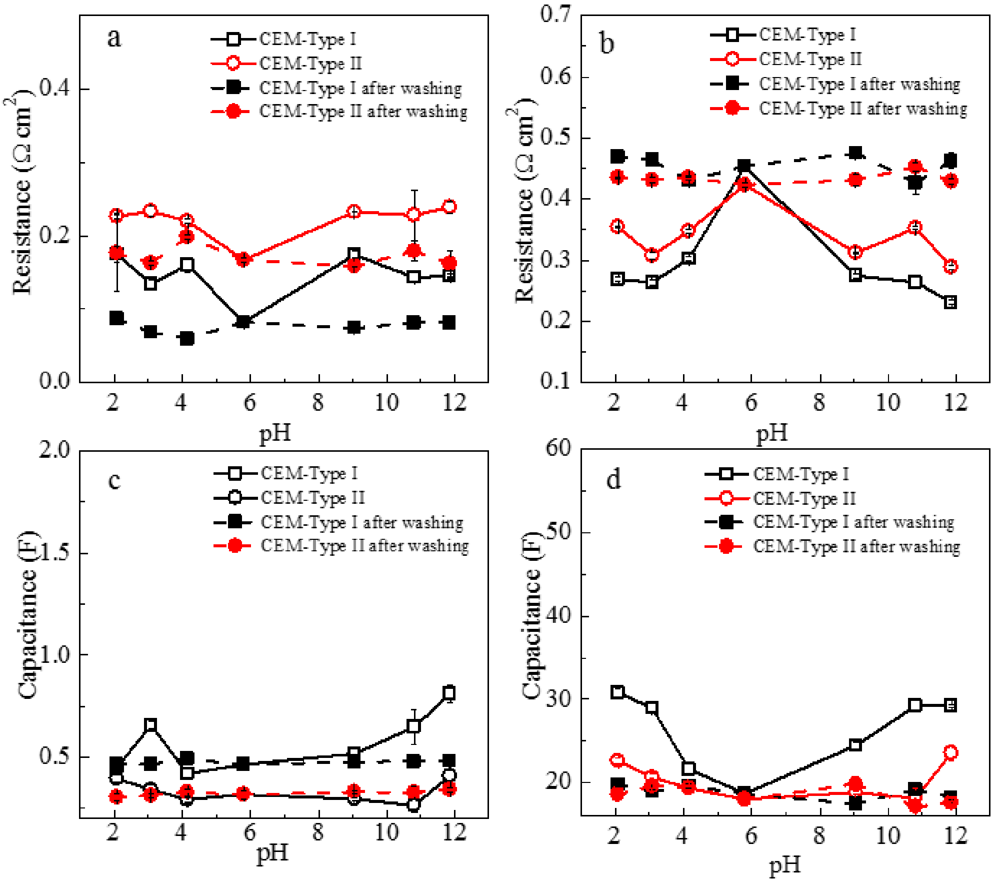

Figure S10. (a) Resistance of EDL (R_{edl}), (b) resistance of DBL (R_d), (c) effective capacitance of EDL (C_{edl}) and (d) effective capacitance of DBL (C_d) in for cation exchange membranes before and after washing with 0.5 mol/L NaCl solution.

References

[1] H. Farrokhzad, M.R. Moghbeli, T. Van Gerven, B. Van der Bruggen, Surface modification of composite ion exchange membranes by polyaniline, Reactive and Functional Polymers 86 (2015) 161-167.

[2] W. Zhang, J. Ma, P. Wang, Z. Wang, F. Shi, H. Liu, Investigations on the interfacial capacitance and the diffusion boundary layer thickness of ion exchange membrane using electrochemical impedance spectroscopy, Journal of Membrane Science 502 (2016) 37-47.

[3] R.K. Nagarale, G.S. Gohil, V.K. Shahi, R. Rangarajan, Preparation and electrochemical characterizations of cation-exchange membranes with different functional groups, Colloids and Surfaces A: Physicochemical and Engineering Aspects 251 (2004) 133-140.

[4] S. Chempath, B.R. Einsla, L.R. Pratt, C.S. Macomber, J.M. Boncella, J.A. Rau, B.S. Pivovar, Mechanism of Tetraalkylammonium Headgroup Degradation in Alkaline Fuel Cell Membranes, The Journal of Physical Chemistry C 112 (2008) 3179-3182.

[5] G. Merle, M. Wessling, K. Nijmeijer, Anion exchange membranes for alkaline fuel cells: A review, Journal of Membrane Science 377 (2011) 1-35.

[6] V. de Paul Nzuwah Nziko, J.-L. Shih, S. Jansone-Popova, V.S. Bryantsev, Quantum Chemical Prediction of pKa Values of Cationic Ion-Exchange Groups in Polymer Electrolyte Membranes, The Journal of Physical Chemistry C 122 (2018) 2490-2501.
